# Research on Performance Evaluation of Polymeric Surfactant Cleaning Gel-Breaking Fluid (GBF) and Its Enhanced Oil Recovery (EOR) Effect

**DOI:** 10.3390/polym16030397

**Published:** 2024-01-31

**Authors:** Yubin Liao, Jicheng Jin, Shenglin Du, Yufei Ren, Qiang Li

**Affiliations:** 1 College of Construction Engineering, Jilin University, Changchun 130026, China; 2 No. 4 Oil Production Plant, PetroChina Qinghai Oilfield Company, Mangya 816400, China; jjcqh@petrochina.com.cn (J.J.); dslbyqh@petrochina.com.cn (S.D.); ryfqh@petrochina.com.cn (Y.R.)

**Keywords:** shale oil, clean fracturing fluids, gel-breaking fluids, reutilization, emulsion, EOR

## Abstract

Clean fracturing fluid has the characteristics of being environmentally friendly and causing little damage to reservoirs. Meanwhile, its backflow gel-breaking fluids (GBFs) can be reutilized as an oil displacement agent. This paper systematically evaluates the feasibility and EOR mechanism of a GBF based on a polymer surfactant as an oil displacement system for reutilization. A rotating interfacial tensiometer and contact angle measuring instrument were used to evaluate the performance of reducing the oil–water interfacial tension (IFT) and to change the rock wettability, respectively. Additionally, a homogeneous apparatus was used to prepare emulsions to evaluate GBF’s emulsifying properties. Finally, core flooding experiments were used to evaluate the EOR effect of GBFs, and the influence rules and main controlling effects of various properties on the EOR were clarified. As the concentration of GBFs increases, the IFT first decreases to the lowest of 0.37 mN/m at 0.20 wt% and then increases and the contact angle of the rock wall decreases from 129° and stabilizes at 42°. Meanwhile, the emulsion droplet size gradually decreases and stabilizes with increases in GBF concentration, and the smallest particle size occurs when the concentration is 0.12–0.15 wt%. The limited adsorption area of the oil–water interface and the long molecular chain are the main reasons that limit the continued IFT reduction and emulsion stability. The oil displacement experiment shows that the concentration of GBF solution to obtain the best EOR effect is 0.15 wt%. At this concentration, the IFT reduction and the emulsification performance are not optimal. This shows that the IFT reduction performance, reservoir wettability change performance, and emulsification performance jointly determine the EOR effect of GBFs. In contrast, the emulsifying performance of GBFs is the main controlling factor for the EOR. Finally, the optimal application concentration of GBFs is 0.15–0.20 wt%, and the optimal injection volume is 0.5 PV.

## 1. Introduction

As conventional oil and gas resources enter the middle and late development stages, the development and utilization of unconventional oil and gas resources such as shale oil, tight oil, and heavy oil have become the main topics of scientific research and engineering construction [1,2,3,4]. The marine shale oil revolution in the United States has promoted the rapid growth of shale oil and gas at an average annual rate of 25% in the past 10 years [5]. However, the development of continental shale oil in China has been more difficult and is still in the development stage [6,7]. Continental shale is mainly formed in semi-deep-water to deep-water lacustrine sedimentary environments. The distribution area of the shale series is small, with diverse lithofacies types and poor structural environment stability [8,9]. Meanwhile, continental shale reservoirs are highly heterogeneous, with well-developed micro- and nano-pores and low development of organic pores, which leads to a complex distribution and small area of the “sweet areas” [10,11]. The above geological characteristics of continental shale oil have led to problems in shale oil development, such as the inability to inject and produce, rapid reduction in natural production capacity, and high precision requirements for production equipment. Hydraulic fracturing is a key production and injection technology for efficient exploitation of shale reservoirs [12,13]. It can connect organic pores through hydraulic fracturing fractures and use proppant to maintain the opening of fractures, greatly improving reservoir permeability and fluid mobility [14,15,16]. Conventional water-based gel-fracturing fluids (slick water, etc.,) have been widely used due to their low friction resistance and strong sand-carrying ability [17]. However, this type of polymer-based gel fracturing fluid produces a large amount of residue, which is an important source of pollution in oil field development as the flowback fluid is produced [18,19]. Developing a clean fracturing fluid system that is safe, environmentally friendly, and reusable while ensuring the basic performance of the fracturing fluid is key to promoting the efficient development of shale reservoirs.

The development and use of clean fracturing fluid originated at the end of the 20th century [20]. It is based on surfactant polymerization to form a viscoelastic surfactant gel as a thickened fracturing fluid system. This type of surfactant is generally a quaternary ammonium salt fatty acid surfactant, which exists as a spherical micelle in water [21,22,23]. When a salt solution is added to the surfactant solution, the spherical micelles further polymerize to form rod-shaped micelles (also called worm micelles), thereby significantly increasing the viscosity of the solution. When the micellar solution encounters oil or gas, it destroys the interaction between the surfactant and the salt and automatically breaks the gel [24,25]. The viscosity of the solution is significantly reduced to facilitate the backflow of the gel-breaking fluids. The cleaning fracturing fluid does not require the addition of chemical agents to form and break the gel, and the residual rate of the flowback solution is almost zero [26]. Therefore, clean fracturing fluid has the advantages of low reservoir damage, low environmental pollution, and single composition of the flowback solution and can be reutilized as an oil-displacement system [22]. Dantas et al. [27] conducted stable shear and oscillatory shear experiments to evaluate the rheological properties of anionic surfactant gel-breaking fluids. He pointed out that small changes in the surfactant will seriously affect the polymerization effect of the micelles and reveal its microscopic mechanisms. Legemah et al. [28] developed a new low-polymer-loaded fracturing fluid containing a boron cross-linking agent, which can significantly improve the sand-carrying capacity of the fracturing fluid while reducing the damage of polymer residues to reservoirs. Chieng et al. [29] improved the traditional single-chain viscoelastic surfactant and proposed a new type of high-temperature-resistant and shear-resistant thickening fracturing fluid. Li et al. [30] pointed out that a large amount of active substances in clean fracturing fluid will reduce the oil–water interfacial tension, change the wettability of the reservoir, and weaken the capillary driving pressure. However, the experimental results found that clean fracturing fluid has a good effect on improving oil recovery, which is attributed to the contribution of osmotic pressure. Meanwhile, Dai et al. also pointed out that osmotic pressure is an additional phenomenon that reduces interfacial tension and is the main controlling factor for gel-breaking fluids to improve oil recovery through studies on the adsorption rules of surfactants at the oil–water rock interface [31,32]. In addition, CO_2_-responsive clean fracturing fluid also has good application prospects [33,34,35].

Additionally, a surfactant is an important chemical agent in tertiary oil recovery [36]. It is usually used in combination with polymers and microspheres or the form of a polymeric surfactant as an oil displacement agent [37,38,39]. It can improve oil recovery by reducing the capillary number, changing reservoir wettability, emulsifying crude oil, and modifying the reservoir profile [40,41,42]. The main component of gel-breaking fluids is the surfactant, which not only plays the role of imbibition and oil recovery during the fracturing backflow process but can also be reutilized as an oil displacement agent in the fracture and pore throat. Wang et al. [43] considered the coupling effects of wettability changes and interfacial tension reduction on surfactant adsorption and revealed the mechanism of wettability changes in different surfactants in carbonate and sandstone reservoirs. The change in reservoir wettability caused by surfactants is considered an important EOR mechanism when injecting liquid into low-permeability shale reservoirs [44,45,46]. Based on micro-pore-scale visualization experimental research, Yekeen et al. [47] explored the synergistic effect of nanoparticles and surfactants in reducing oil–water interfacial tension, changing rock wettability, and stabilizing emulsion. He pointed out that nano-surfactants show a good tendency to significantly reduce the oil–water interfacial tension and change the rock-wetting properties [48,49,50,51,52].

The above-mentioned research found that surfactant-based clean fracturing fluid systems are beneficial to replace traditional thickened gel fracturing fluids and can be reutilized as oil displacement systems after the fracturing fluids flow back. The current research has the following three problems: (1) the current clean fracturing fluid systems are all based on low-molecular surfactants, and there is a lack of research on clean fracturing fluid systems based on long-chain polymer surfactants; (2) at present, the synthesis of clean fracturing fluids only pursues the properties of ultra-low interfacial tension and ignores the main control effect of the emulsification performance of the gel-breaking fluid on the EOR; (3) most research on the EOR mechanisms of gel-breaking fluids are aimed at the imbibition process, lacking the EOR potential of reducing interfacial tension, wettability modification, and emulsification in its reutilized process.

Therefore, based on the above problems, this paper conducts experimental research on a clean fracturing fluid and its gel-breaking fluid based on polymer surfactants. The interfacial tension reduction performance, wettability improvement performance, and emulsification performance were evaluated, and the influence of concentration on the above properties was clarified. Finally, the relationship between the EOR and corresponding properties under different gel-breaking fluid concentrations is compared, and the EOR mechanism and the main control role of each property in its reutilized process are explained. Finally, the gel formation, gel breaking, and interfacial adsorption processes of cleaning fracture fluids are illustrated through illustrations. This paper highlights that under non-ultra-low interfacial tension, gel-breaking fluids can give full play to the main control role of emulsification in improving oil recovery and have good potential for oil displacement and reutilization.

## 2. Material and Method

### 2.1. Materials

Raw materials: Dichloromethane and ethanol used for core oil washing were purchased from KeLong Chemical Co., Ltd. (Chengdu, China); Inorganic salts such as NaCl, KCl, CaCl_2_, etc., were purchased from Aladdin Biochemical Technology Co., Ltd. (Shanghai, China); Ethylene glycol monobutyl ether used for preparing the breaking fluids was purchased from Winson New Material Technology Co., Ltd. (Shanghai, China). Deionized water (DI water) was prepared by our lab using one comprehensive deionized water ultrapure water machine (WPL-EDI-DI-UP-10-UVF, Shanghai, China).

Clean fracturing fluids (CFF): CFF is mainly composed of a cationic viscoelastic polymer surfactant (VEPS), whose main component is long-chain tertiary amine (LCTA). By configuring aqueous solutions with LCTA and KCl at a mass ratio of 3 wt% and 4 wt%, respectively, the clean fracturing fluid CFF with good suspension properties was obtained, with a viscosity of approximately 120 cP at 70 °C.

Gel-breaking clean fracturing fluids (GBFs): For the GBFs, 1% kerosene was added to the CFF solution, which was stirred evenly at 25 °C for about 90 min until the gel broke. After the system was fully stable, we used a centrifuge to separate the kerosene to obtain the required gel-breaking fluid. The quality of the solution remained unchanged during the preparation process of the CFF and GBF, so the effective concentration of LCTA in the GBF solution was 3 wt%.

Solution: The degassed and dehydrated crude oil came from the Changqing Oilfield in China, and its viscosity and density at 70 °C were 1.34 cP and 0.734 g/cm^3^, respectively. The simulated formation water was prepared by sequentially adding inorganic salts to DI water according to the formula in Table 1 (the total salinity was 37,683 mg/L).

Cores: The cores were natural cylindrical cores obtained in the laboratory from the Changqing Oilfield (diameter was 2.5 cm, length was 5–10 cm, and permeability was within 5 mD). There was crude oil in the natural cores, which needed to be removed using ethanol and dichloromethane (DY-4 core rapid oil washing instrument, Yangzhou, China). The natural cores were placed in an oven to dry after oil washing for 3 days, and then the dry weight was recorded as m_1_. The detailed parameters of the cores used in Section 2.2.4 are shown in Table 2.

### 2.2. Methods

#### 2.2.1. Evaluation of Interfacial Tension

Reducing the oil–water interfacial tension is a basic property of clean fracturing fluid, which can increase oil recovery by imbibition of the fracturing fluid matrix, making the crude oil easier to remove. A rotating interfacial tensiometer (Texas-500-C, 10^2^–10^−5^ mN/m, Kono, Seattle, WA, USA) was used to measure the interfacial tension between CFF solution with different concentrations and dehydrated crude oil under 80 °C. To ensure the accuracy of the experimental results, a parallel control experiment was conducted for each group of IFT test experiments.

The specific test steps are: (1) Take the GBF solution prepared in the Materials section and dilute it with simulated formation water to concentrations of 0.02 wt%, 0.05 wt%, 0.08 wt%, 0.12 wt%, 0.15 wt%, and 0.20 wt%, 0.25 wt%, 0.30 wt%. (2) Use a needle to take a GBF solution and fill it into the interfacial tension meter test tube, being careful not to retain bubbles. (3) Use another special needle to absorb the crude oil and squeeze it into the solution in the tube. The main oil droplets (about 0.1 mL) should be of moderate size and should not be in contact with the wall of the test tube. (4) Turn on the temperature control system of the interfacial tension meter and wait for 2 h of constant temperature before testing. (5) Turn on the rotating interfacial tension meter, rotate the test tube at 3000 rpm, and use the image acquisition system to record the shape of the oil droplet in real time. (6) When the shape of the oil droplet does not change, take a photo to record the shape of the oil droplet and use the software analysis system to calculate the oil–water interfacial tension. (7) Stop rotating the interfacial tension meter, take out, clean, and dry the test tube. (8) Use GBF solutions of other concentrations and repeat steps (2) to (7) to obtain the oil–water interfacial tension under different GBF solution concentrations.

#### 2.2.2. Evaluation of Wettability

The pore throats of shale reservoirs are small, and the lipophilic characteristics of the rock surface cause an oil film to be adsorbed on the surface, which not only reduces the oil-washing efficiency but also reduces the effective flow radius of the pore throats, exacerbating the difficulty of oil development. When the reservoir is oil wet, the contact angle of the solution is greater than 90°. Here, a static-contact goniometer (DSA100, Kruss, Hasvink, Germany) is used to test the effect of different concentrations of GBF solutions on rock wettability. The contact angle test software is equipped with an analysis system. Here, considering the contact angle range, the goniometric method is used to automatically measure and calculate the contact angle.

The specific test steps are: (1) Place several natural core sample slices and crude oil in a Petri dish at a volume ratio of 1:4 and age them for 1 day to obtain strongly lipophilic core slices. (2) Take the GBF solution prepared in the Materials section and dilute it with simulated formation water to concentrations ranging from 0.05 and 0.3 wt%. (3) Turn on the contact angle tester, and use a needle to absorb the GBF solution and drop it on the core slice (about 0.1 mL). (4) Place the core slice on the test bench, take pictures and record the images when the droplet shape does not change, and use the processing system to calculate the contact angle. (5) Replace the core slices and GBF solution and repeat step (3) and step (4) to obtain the contact angles of solutions with different GBF concentrations. As a baseline, the contact angle of DI water is 129.47°.

#### 2.2.3. Evaluation of Emulsion Ability

The emulsifying property of GBF is key to its role in improving oil recovery. The evaluation of emulsifying properties usually includes emulsification strength and emulsion stability. The emulsion strength is evaluated by the emulsion droplet size distribution curve. The smaller the emulsion droplet size and the more uniform the distribution, the higher the emulsification strength. The stability of the emulsion is evaluated by the change curve of the liquid separation rate of the emulsion after standing. The faster and more complete the delamination, the worse stability. Here, a dispersion homogenizer (FJ300-S digital, Youyi, Shanghai, China) was used to prepare an emulsion of GBF solution and crude oil, and the particle size distribution was observed using a stereomicroscope (XTL-7000C, Caikon, Shanghai, China).

The specific experimental steps are: (1) Prepare 20 mL of GBF solution with a concentration of 0.05 wt%, 0.08 wt%, 0.12 wt%, 0.15 wt%, and 0.20 wt%. (2) Weigh 10 mL GBF solution and 10 mL crude oil, respectively, into a 30 mL beaker. (3) Use a homogenizer to mix the solution at a speed of 2000 rpm for 1 min and then pour it into a 25 mL test tube. (4) Place the test tube in an 80 °C incubator and record the amount of water precipitated in the test tube at certain intervals. We defined the ratio of the volume of precipitated water in the test tube recorded each time to the volume of added water (i.e., 10 mL) as the liquid drainage rate. Drawing the curve of the liquid drainage rate over time can clarify the stability of the emulsion. (5) Repeat step (3) to obtain the mixed emulsion, and draw a sample from the middle of the solution and drop it on a glass slide to observe its particle size using a stereomicroscope. (6) Replace the GBF solution and repeat steps (2) to (5) to evaluate the emulsion properties of solutions with different GBF concentrations.

#### 2.2.4. Core Displacement Experiments

The reduction in interfacial tension, change in wettability, and emulsification ability of the GBF solution directly affect its final EOR effect. Meanwhile, the injection amount of the GBF solution will also affect its efficiency. Here, the influence of various parameters on the EOR effect of GBFs is evaluated through core flooding experiments. The specific experimental plan is shown in Table 3. The EOR effect of GBF flooding is the difference between the final oil recovery and the water flooding oil recovery. In order to ensure the accuracy of the experimental results, a parallel control experiment was conducted for each displacement experiment.

**Figure 1:**
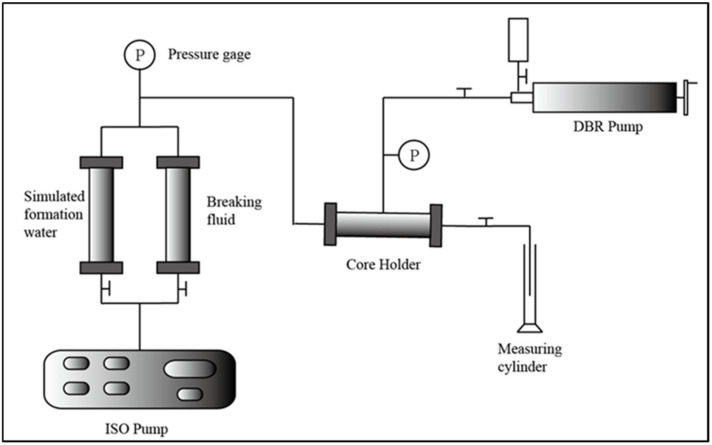
The flowchart of the displacement process.

The specific experimental steps are: (1) Put the core into the core holder and use a vacuum pump to evacuate the core end 1 for 4 h. (2) Saturate the simulated formation water. Connect end 2 of the core holder into a beaker containing simulated formation water and open the valve to self-absorb saturated water for 2 h. Record the volume change in the simulated formation water in the beaker before and after self-priming, which is the core pore volume V_1_. Additionally, take out the saturated core and remove the surface water stains to weigh the wet weight m_2_. Then, calculate the saturated water mass as m_1_-m_2_ and calculate the pore volume V_2_ based on the simulated formation water density. Use V_1_ and V_2_ to correct the pore volume as V = (V_1_ + V_2_)/2 and calculate the porosity. (3) Absolute permeability test. Connect the experimental process according to Figure 1; use a constant speed pump to inject simulated formation water at a constant rate of 0.1 mL/min, and record the injection pressure. After recording the stable injection pressure, Darcy’s law is used to calculate the core permeability. (4) Saturated crude oil. Use a constant speed pump to inject crude oil at a constant rate of 0.05 mL/min until no water is produced at the production end. Increase the injection rate to 0.2 mL/min and continue to inject 1 PV of crude oil. Stop the injection and place it in an 80 °C oven for aging for 3 days. Record the volume of produced water as the saturated oil volume and calculate the oil saturation S_o_. (5) Carry out the oil displacement experiment according to the plan in Table 3 and record the injection pressure and liquid production throughout the process. Then, you calculate water cut (percentage of produced water volume to produced liquid volume) and oil recovery factor (percentage of produced oil volume to saturated oil volume) to compare their changes overtime. Keep the experimental temperature kept at 80 °C using one thermostat, and carry out the displacement experiments at a constant injection rate of 0.1 mL/min.

## 3. Results and Discussion

### 3.1. IFT Reduction Ability of GBF

**Figure 2:**
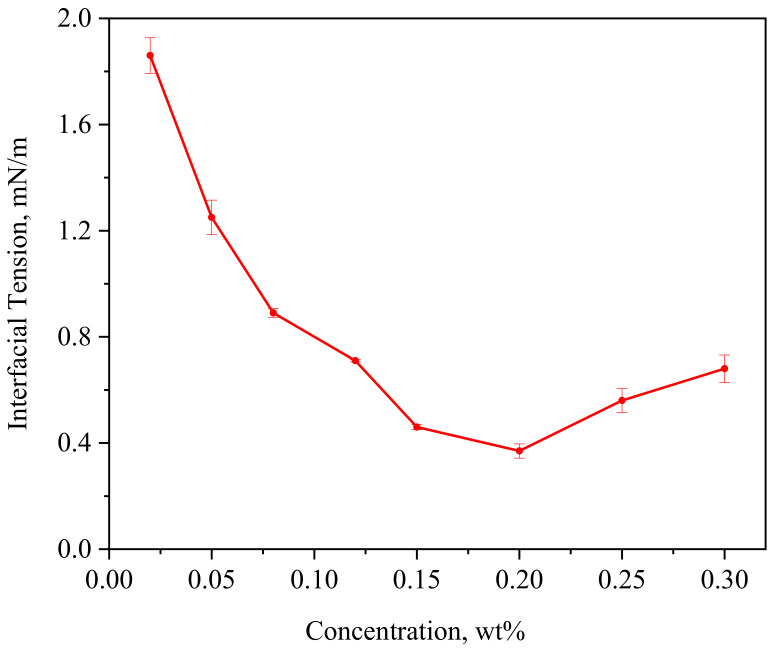
The steady-state IFT between crude oil and GBF with different concentration (the IFT between crude oil and DI water is 25.42 mN/m).

After fracturing in shale reservoirs, the clean fracturing fluid will break through contact with crude oil and become a GBF. The GBF can fully contact the crude oil and wall surface in the reservoir, and the decrease in IFT can lead to the decrease in the adhesion energy, making the crude oil more easily peeled off from the rock surface. The curve of IFT response to the GBF concentration is shown in Figure 2.

The steady-state IFT between crude oil and the GBF first decreases rapidly with the increase in GBF concentration, reaching the lowest value of 0.37 mN/m when the concentration is 0.2 wt%. Then, as the GBF concentration continues to increase, the IFT increases slowly. The CFF system used in this paper is a polymeric surfactant gel. The molecular chain of this surfactant is significantly larger than that of conventional surfactants, so its ability to reduce IFT is limited. This type of surfactant has a similar EOR mechanism to the polymeric surfactant and can achieve a good oil-washing effect at a relatively low IFT (the oil–water IFT test result is 34.26 mN/m). As the GBF concentration increases, there are two main reasons for the upward trend of IFT: (1) The oil–water interface area is limited, and continuing to increase the concentration of active agent molecules cannot increase the effective adsorption capacity. Meanwhile, the GBF is a long-chain molecule, which will reduce the adsorption area of active groups after adsorption at the interface. (2) GBF is a gel-breaking solution of CFF. When the concentration of the GBF gradually increases, it is easy to form a micelle gel again, thereby reducing the interfacial adsorption of the GBF.

In addition, it can be found that as the GBF concentration increases, the error of IFT first gradually decreases and then increases again. This is also related to the interface adsorption of GBF molecules. When the GBF concentration is low or high, the adsorption number of molecules at the interface will be affected by the solution preparation process, resulting in fluctuations in IFT test results.

### 3.2. Wettability Alteration Ability of GBF

**Figure 3:**
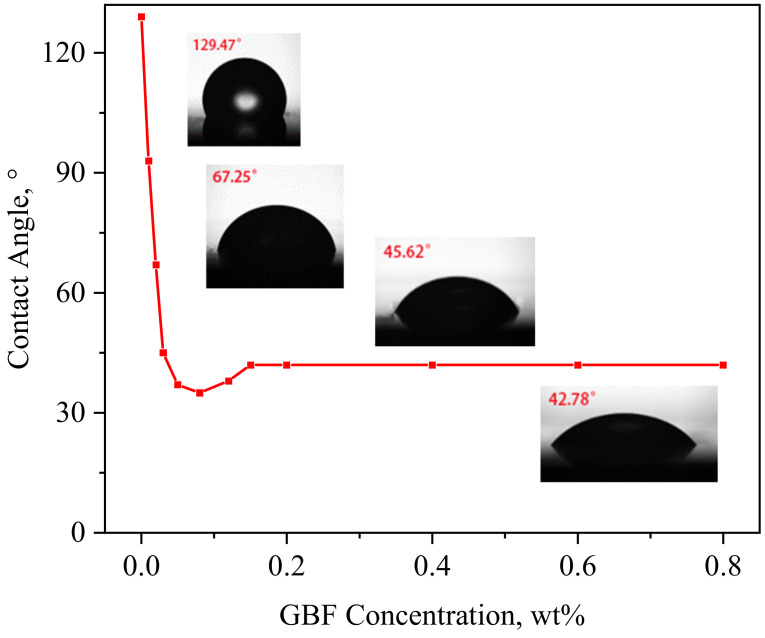
Variation curve of contact angle of oil-wet core thin sections with GBF concentration (the contact angle of DI water is 129.47°).

The GBF solution can strip crude oil by reducing the oil–water IFT; in addition, the oil displacement efficiency is closely related to the wettability of the rock. The oil-wet surface causes the oil displacement efficiency to deteriorate, but the water-wet surface can increase the oil displacement efficiency. Therefore, the change in reservoir wettability is another main EOR mechanism of the GBF solution. The change curve of contact angle with the GBF solution concentration is shown in Figure 3.

It can be seen from Figure 3 that the wetting angle decreases sharply with the increase in the GBF concentration within 0.1 wt% and then stays stable with the further increase in concentration. The reason is that GBF molecules are adsorbed on the oil-wet quartz surface through electrostatic attraction, Van Der Waals force, hydrogen bond, and hydrophobic action. The surfactant molecule hydrophobic group assembles directly with a solid surface and the hydrophilic group makes contact with an aqueous solution, which reduces the interfacial energy, and adhesion tension increases, s o the contact angle steeply falls, leading to enhanced water wettability on the surface of the quartz plate.

As the GBF concentration further increases, the adsorption capacity of the surfactant molecules increases, and the adsorption of surfactant molecules on the surface of quartz can cause a decrease in electronegativity. When the GBF concentration reaches a certain value, the interfacial energy and adhesion tension basically remain the same, so the contact angle tends to be stable at 42°. This suggests that the GBF solution can change the quartz surface from oil-wet to water-wet, which, combined with the reduction in oil–water IFT, greatly improves the oil-washing efficiency of shale reservoirs.

### 3.3. Emulsification Property of GBF

The GBF solution strips crude oil by reducing the IFT and changing rock wettability, which is the first step in improving oil recovery. The second step is to emulsify the stripped crude oil into an interfacial energy minimization system in which oil and water are evenly dispersed so that it can be easily carried and extracted in the form of emulsion droplets.

**Figure 4:**
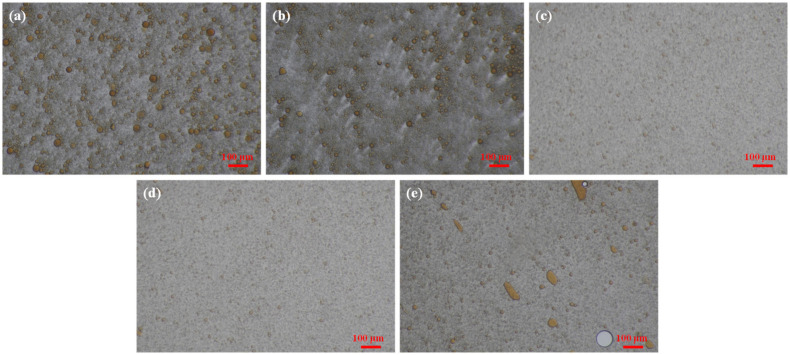
The droplet size of GBF emulsion with different concentrations. (**a**) GBF concentration is 0.05 wt%; (**b**) GBF concentration is 0.08 wt%; (**c**) GBF concentration is 0.12 wt%; (**d**) GBF concentration is 0.15 wt%; (**e**) GBF concentration is 0.20 wt%.

Figure 4 is the microscopic morphology of the droplet size distribution after crude oil is emulsified with GBF solutions of different concentrations. It can be found that as the concentration of the GBF solution increases, the emulsion droplet size first gradually decreases and then remains stable. It can be clearly observed that the granularity of the emulsion droplets has become weaker, which is the result of the high dispersion of the emulsion droplets. When the GBF solution concentration is 0.2 wt%, although the particle size of the emulsion droplets does not increase significantly, there are some large oil lumps, which have a serious impact on its stability.

**Figure 5:**
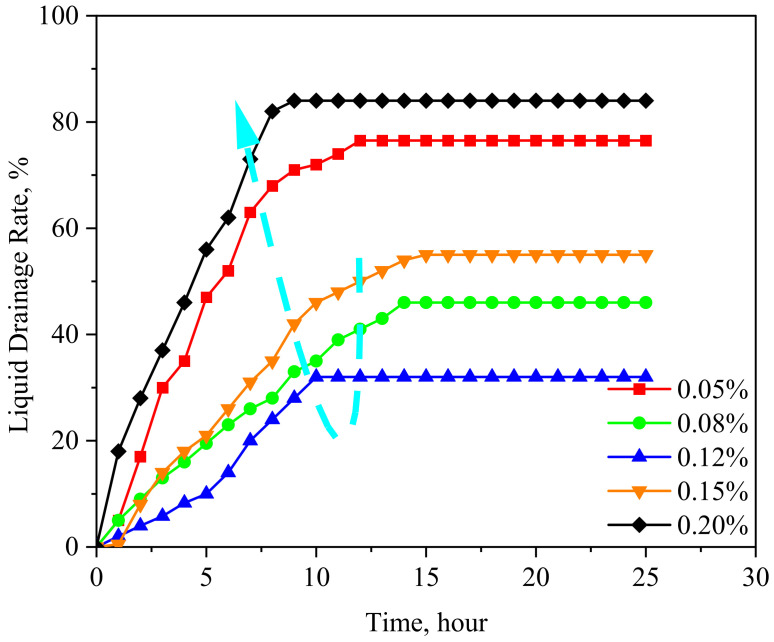
The water drainage rate of GBF change curves.

The liquid separation processes of the GBF emulsion with different concentrations are shown in Figure 5. It can be seen that the water drainage rate of the emulsion increases with the aging time and then stays stable. The liquid separation process of the GBF emulsion can last for 10–15 h, which is enough to illustrate its good stability. The changing trend of the final liquid drainage rate is that it first decreases and then increases with the increase in GBF concentration (as shown in the cyan dotted line in Figure 5). When the GBF concentration is 0.12 wt%, the liquid drainage rate is the lowest at 32%, and there is still a large amount of emulsion in the upper solution. When the GBF solution concentration is 0.20 wt%, the liquid drainage rate reaches a maximum of 85%. The change law of liquid drainage rate with the GBF concentration is consistent with its IFT reduction rule, and their mechanism of action is also the same. The decisive factor of the emulsion stability is the strength of the interface membrane. When the GBF concentration is small, fewer molecules are irregularly adsorbed on the interface and the membrane strength is weakening. So, the emulsion system is easy to demulsify and dehydrate with poor emulsion stability. When the GBF concentration is extremely large, the formation of micelles will damage the interface membrane, resulting in an increase in the water drainage rate.

GBF strips crude oil and emulsifies it for extraction, which increases its oil-washing efficiency. Meanwhile, the emulsion can coalesce to form an oil band, and the oil band continues to encounter dispersed crude oil as it moves forward. The oil band formed by emulsified crude oil in the high permeability zone can modify the injection profile because of the Jamin effect. Thus, the GBF solution can also expand the swept volume until the oil band is taken out.

GBF is a potential EOR technology which has two EOR mechanisms: expanding the swept volume and improving the oil washing efficiency. Through the evaluation of the IFT reduction performance, wettability change performance, and emulsification performance, it can be determined that the optimal application concentration of GBF is 0.15 wt%. Its EOR efficiency will be evaluated in Section 3.4.

### 3.4. EOR Effect of GBF

Seven sets of GBF oil displacement experiments were carried out according to Table 3. The first six experiments of fixed injection volume were 0.3 PV to evaluate the impact of GBF concentration on the EOR effect. Meanwhile, the impact of IFT-reducing ability, wettability-changing ability, and emulsifying ability on the EOR at the corresponding concentration was evaluated. The seventh experiment fixed the GBF concentration at 0.15 wt% and performed a continuous GBF injection of 1.2 PV. During the period, the EOR at different injection volumes was intercepted to evaluate the impact of the GBF injection amount on the EOR effect.

**Figure 6:**
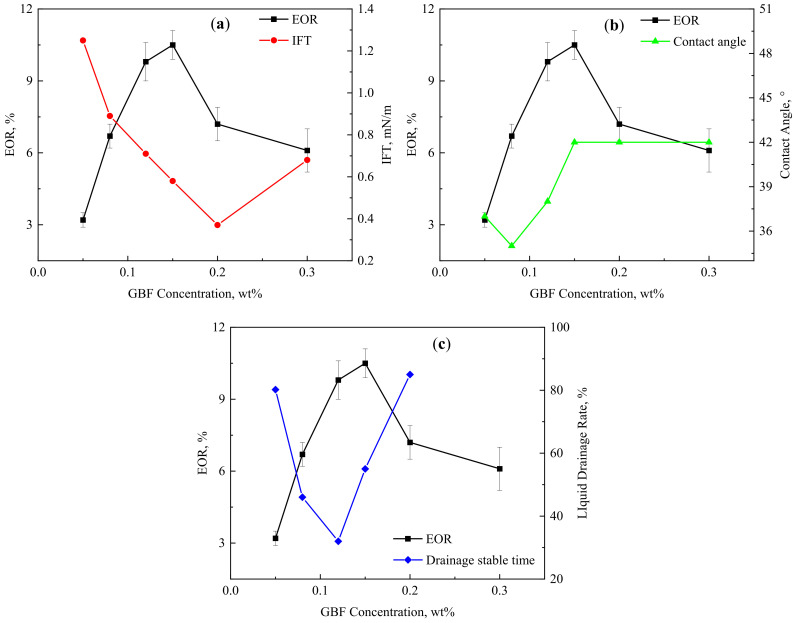
EOR and corresponding interface characteristic curves of GBF solutions at different concentrations. (**a)** IFT; (**b**) contact angle; (**c**) drainage stable time.

The EOR and corresponding IFT, contact angle, and emulsion drainage stabilization time curves under six GBF concentrations are shown in Figure 6. The error bar of the EOR shows that the results of each experiment fluctuate within a certain range, but it can be found that this does not affect the change rule of EOR with GBF concentration. The main reasons for the error are the influence of experimental operations and the small differences in core parameters. The existence of the error bars proves the reliability of the experimental results and the objectivity of the rules. The EOR first increased significantly with the increase in GBF concentration and then showed a downward trend. The best EOR effect occurs when the GBF concentration is 0.15 wt%. Figure 6a shows that the concentration of the GBF solution with the lowest IFT is 0.20 wt%, and the EOR effect at this time begins to decrease. This is mainly because the stability of the GBF emulsion decreased significantly at this time, as shown in Figure 6c. Figure 6b shows that within the test concentration range, the contact angle is at a low value and tends to be stable, and its change pattern does not directly correspond to the EOR change rule. Figure 6c shows that the GBF concentration that obtains the best EOR is 0.15 wt%, which is inconsistent with the concentration corresponding to the lowest IFT and the lowest liquid drainage rate. This illustrates that the interfacial tension reduction performance and emulsification stability jointly determine the EOR effect of the GBF. However, the EOR when the liquid drainage rate is the lowest is closest to the optimal EOR. Overall, the increase and decrease in the EOR curve corresponds to the decrease and increase trend of the liquid drainage rate curve, which shows that among the three properties, the stability of the GBF emulsion is the main decisive factor controlling its EOR effect. Therefore, pursuing only ultra-low levels of IFT is not the correct direction for future oil displacement system construction.

**Figure 7:**
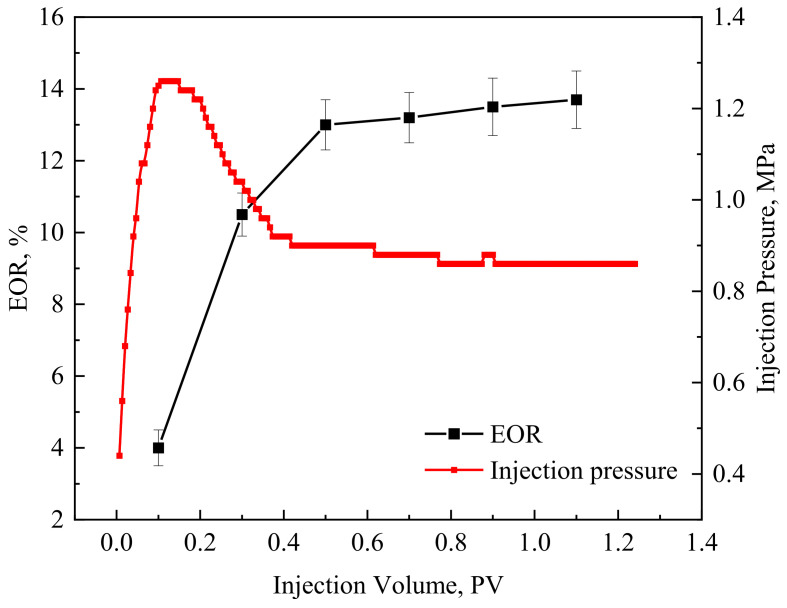
The relationship between the injection of slug and the growth rate of recovery.

The injection pressure will increase significantly after the GBF solution is injected. Although GBF can reduce the oil–water IFT to reduce pressure and increase injection, the emulsion formed at the same time will produce a Jamin effect at the pore throat and increase the injection pressure. The GBF injection pressure curves and corresponding EOR effects under different injection volumes are shown in Figure 7.

Figure 7 shows that the injection pressure of the GBF stabilizes when the injection volume reaches 0.2 PV. At this time, the oil wall corresponding to the emulsion aggregation is produced. The EOR effect is significantly improved at this stage. After that, the injection pressure decreases rapidly and eventually levels off. It can be found that the moment when the EOR and the injection pressure become stable is when the injection volume is 0.5 PV, which shows that the increase in injection pressure is the main contribution to the improvement of the EOR effect. The experimental results also illustrate once again that the emulsifying performance of the GBF is the main controlling factor of its EOR efficiency.

Taking into account the various static properties and EOR effects of GBF, the optimal application concentration is 0.12–0.20 wt%, and the optimal injection volume is 0.5 PV. As an oil displacement agent for fracturing fluid reutilization, GBF is compared with a separate oil displacement system and other gel-breaking fluid, as shown in Table 4. It can be found that the ability of GBF to reduce interfacial tension is equivalent to that of a polymeric surfactant, but its viscosity is much lower, so the EOR effect is poor. Additionally, although the compound surfactant can achieve ultra-low interfacial tension and its EOR is higher than that of GBFs, its individual application is rare and the cost increases significantly. Although GBF does not have the advantages of ultra-low interfacial tension and high viscosity of surfactants and a polymeric surfactant, it takes into account the advantages of both. Compared with the EOR of about 10% of ordinary polymers, GBF has considerable EOR capabilities when reutilized as an oil displacement agent and has obvious economic advantages over surfactants.

### 3.5. Mechanism of GBF Formed Process and EOR Effect

Gel-fracturing fluid processes such as CO_2_ response [55], temperature response [56], ion response [29], etc., have the advantages of low viscosity during injection and easy gel breakage and are the development trend of clean fracturing fluid in the future. The clean gel fracturing fluid used in this paper is an ion-responsive polymer micelle gel. Current research has clarified its gel formation, gel breaking, and oil-displacement mechanisms, but there is a lack of comprehensive revelation and description of the above mechanisms. Based on the above experimental results and previous research experience, this paper summarizes the entire process mechanism of clean fracturing fluid, from gel formation to gel breaking and interface action to improve oil recovery.

**Figure 8:**
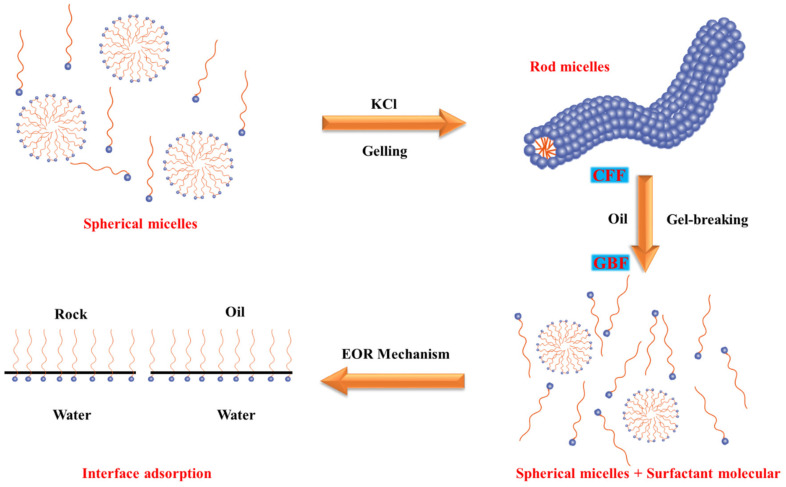
The process of clean fracturing fluid forming gels and breaking gels and the EOR mechanism.

A schematic diagram of the gel formation process of CFF, the gel breaking process to form GBF, and the adsorption mechanism of GBF at the interface, are shown in Figure 8. CFF is composed of a viscoelastic surfactant. In aqueous solutions, the nonpolar groups of these molecules tend to aggregate to avoid contact with water, eventually forming self-assembled spherical micelles. When the concentration of water anions increases, a large number of surfactant molecules further aggregate to form polymeric rod-like micelles, and the mutual entanglement between micelles greatly increases the viscoelasticity of the system. The viscoelasticity of the CFF solution is the basis for its significant sand-carrying performance. When organic matter or other hydrophobic substances (oil or gas) are dissolved in micelles, these rod-shaped micelles will be expanded and dispersed into smaller spherical micelles, resulting in reduced viscosity of the system. As the gel breaking has no breaking agent added, it will not cause any damage to the viscoelastic surfactant molecule and will provide a solid foundation for its further re-utilization.

In the application of the GBF flooding process, the surfactant molecules move toward and adsorb the oil–water interface and rock surface, effectively reducing the oil–water IFT and changing the wettability of the reservoir, which is of great significance to improving oil recovery.

## 4. Conclusions

This paper studies the reuse effect of gel-breaking fluids (GBFs) of a clean fracturing fluid system (CFF) based on polymer surfactants. The interfacial tension reduction performance, wettability change performance, and emulsification performance of GBFs were evaluated, and the main control effect of each factor on the EOR was clarified by comparing the EOR effect under various static properties, and the optimal application parameters were formed. Finally, the gelation and gel-breaking processes of CFF and the interfacial adsorption mechanism of the GBF solution were explained. The specific conclusions are as follows:

(1) GBFs can reduce the oil–water IFT to 10^−1^ mN/m. As the concentration of GBF increases, the oil–water IFT first decreases and then increases. The lowest IFT of 0.37 mN/m occurs when the GBF concentration is 0.20 wt%. The limited adsorption area of the oil–water interface and the long molecular chain are the main reasons that limit the continued IFT reduction.
(2) GBFs can effectively improve reservoir wettability. As the concentration of GBF solution increases, the contact angle of the rock wall decreases from 129° and stabilizes at 42°. Reducing the oil–water IFT and changing the wettability of the reservoir are the fundamental reasons why GBFs can effectively strip crude oil from shale reservoirs.
(3) GBFs have good emulsifying properties. As the concentration of GBF solution increases, the emulsion droplet size gradually decreases and stabilizes, with the smallest particle size at a concentration of 0.12–0.15 wt%. At a concentration of 0.20 wt%, a larger area of oil block will appear, which also corresponds to a significant reduction in emulsion stability.
(4) The optimal application concentration of GBFs is 0.12–0.20 wt%, and the optimal injection volume is 0.5 PV. The oil displacement experiment shows that the concentration of GBF solution to obtain the best EOR effect is 0.15 wt%. At this concentration, the IFT reduction and the emulsification performance are not optimal. This shows that the IFT reduction performance, reservoir wettability change performance, and emulsification performance jointly determine the EOR effect of GBFs. In contrast, the emulsifying performance of GBFs is the main controlling factor for the EOR.
(5) The experimental results of this paper prove that the BGF after breaking the CFF with a polymer surfactant as the main body has good EOR potential. It can effectively improve the oil washing efficiency and expand the swept volume at a lower dosage. Additionally, its main mechanism of action is emulsification rather than ultra-low interfacial tension, which also provides new thinking for the synthesis direction of oilfield chemicals.

## Figures and Tables

**Table 1:** The composition of the simulated formation water.

Iron	K^+^, Na^+^	Ca^2+^	Mg^2+^	CO_3_^2−^	HCO_3_^−^	SO_4_^2−^	Cl^−^	Total
Concentration, mg/L	12,429.68	1110.17	637.88	57.12	1252.12	342.86	21,853.51	37,683

**Table 2:** Basic core parameters.

Core	Pore Volume, mL	Diameter, cm	Length, cm	Permeability, mD	Volume of Oil, mL	Oil Saturation, %
C1	6.8	2.5	7.1	7.5	4.9	72.06
C2	7.1	2.5	7.2	8.3	5.2	73.24
C3	6.3	2.5	6.8	7.2	4.5	71.43
C4	6.5	2.5	6.9	7.5	4.7	72.31
C5	6.7	2.5	7.0	7.7	4.9	73.13
C6	6.7	2.5	7.1	7.8	4.7	70.15
C7	6.9	2.5	7.1	7.9	4.9	71.01

**Table 3:** Oil displacement experimental plan.

Number	Core	GBF Concentration, wt%	GBF Volume, PV	Injection Process	Note
1	C1	0.05	0.3	Water flooding to the water cut reaches 90%—GBF flooding to designed volume	Compare the influence of GBF concentration
2	C2	0.08	0.3		
3	C3	0.12	0.3		
4	C4	0.15	0.3		
5	C5	0.20	0.3		
6	C6	0.30	0.3		
7	C7	0.15	1.2		Compare the influence of GBF volume

**Table 4:** EOR comparison between GBF and other displacement agents.

Number	Agent	Concentration, wt%	IFT, mN/m	Volume, PV	Recovery, %	EOR, %
0	Gel-breaking fluid	0.15	0.46	0.5	58.32	13.00
1 [30]	Gel-breaking fluid	0.70	0.369	Imbibition	33.20	/
2 [31]	Gel-breaking fluid	2.00	0.14	Imbibition	40.00	/
3 [39]	Polymeric surfactant	1.50	~0.90	0.8	/	17.49
4 [53]	Compound surfactant	/	10^−2^	3	/	13.65
5 [53]	Compound surfactant	/	10^−3^	3	/	16.28
6 [54]	ASP (Alkali + Surfactant + Polymer)	S is 0.1–0.3	~10^−2^	0.5–0.8	/	18–28

## Data Availability

Data are contained within the article.
